# Rupture of the Uterus—Gastrotomy

**Published:** 1871-03

**Authors:** 


RUPTURE OF THE UTERUS—GASTROTOMY.—D. J. H. Tylecote records (*Lancet*, Nov. 5, 1870) a case of rupture of the uterus. The child escaped into the abdominal cavity; gastrotomy was performed, and a still-born child removed. The mother recovered.

